# Swallowed by the Past: Dysphagia as a Decade-Late Manifestation of Renal Cell Carcinoma Recurrence

**DOI:** 10.14309/crj.0000000000001693

**Published:** 2025-05-05

**Authors:** Hassam Ali, Fnu Poonam, Prashant Mudireddy

**Affiliations:** 1 Department of Gastroenterology, East Carolina University, Brody School of Medicine, Greenville, NC; 2 Department of Pathology, East Carolina University, Brody School of Medicine, Greenville, NC

## CASE REPORT

**Figure 1.**
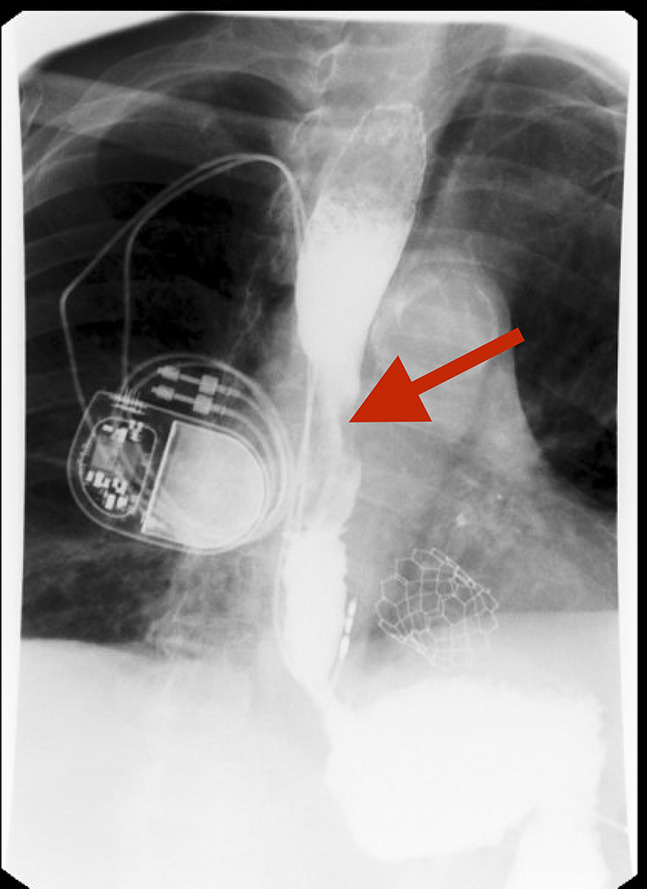
Esophagram demonstrating significant esophageal narrowing. Figure shows an upper gastrointestinal contrast study revealing a tight narrowing of the midesophagus with extrinsic compression (red arrow), suggestive of a mass effect.

**Figure 2.**
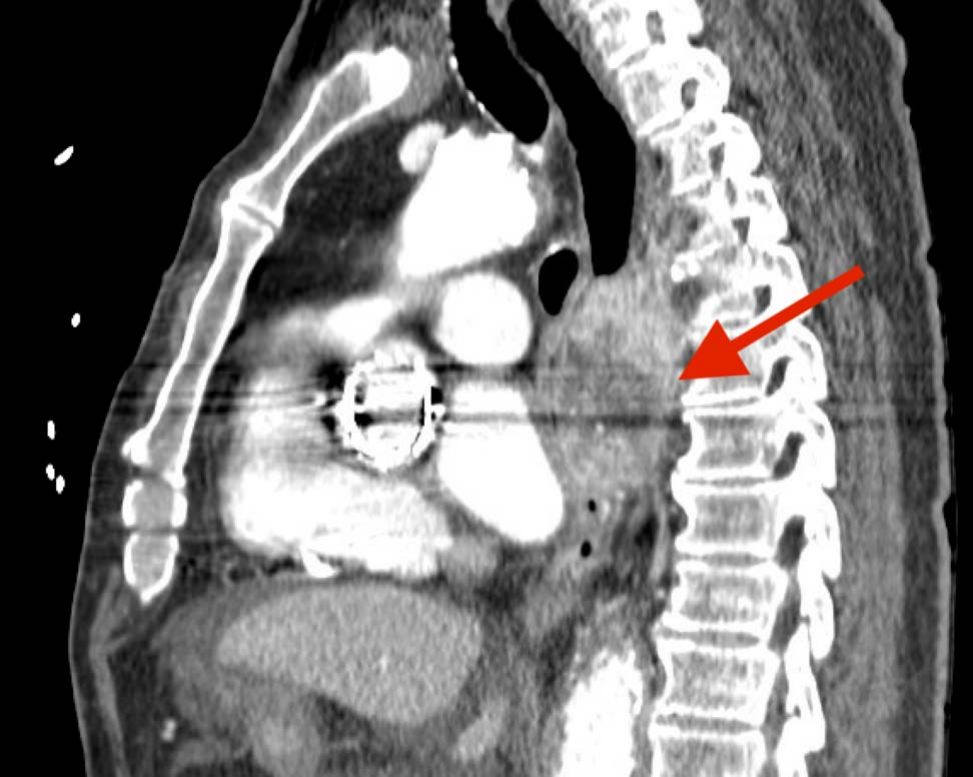
Sagittal view of the chest by contrast-enhanced computed tomography scan. The figure highlights an esophageal mass causing significant luminal obstruction and compression of adjacent structures (red arrow).

**Figure 3.**
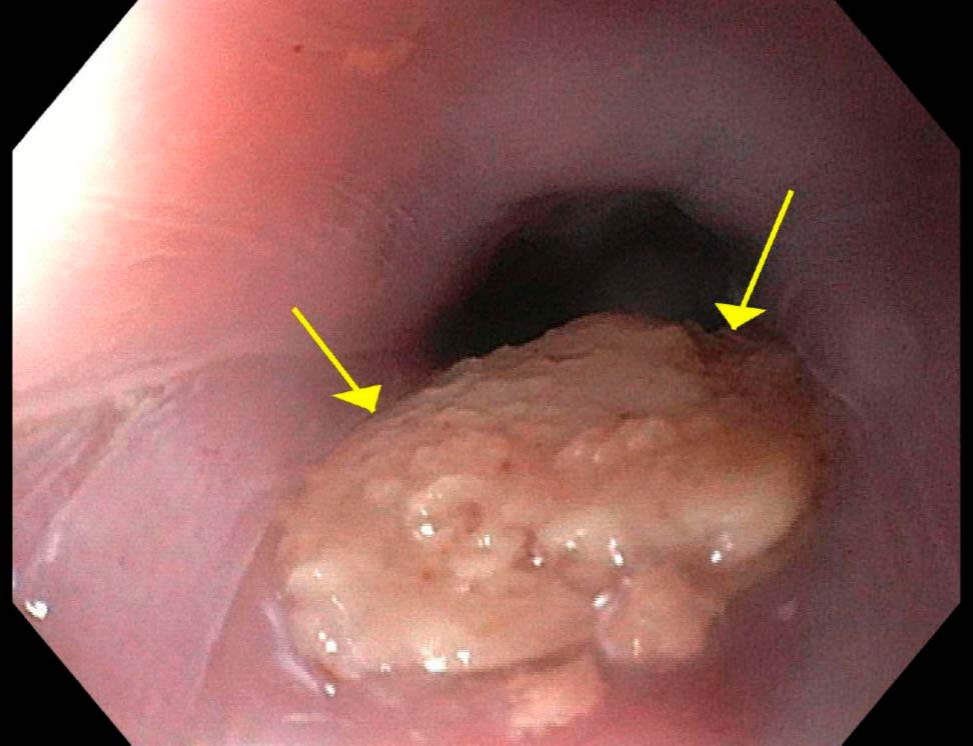
Endoscopic visualization of the esophageal lumen. The figure reveals the intraluminal impression of a smooth, bulky mass (yellow arrows), corresponding to the extrinsic compression noted on imaging studies.

**Figure 4.**
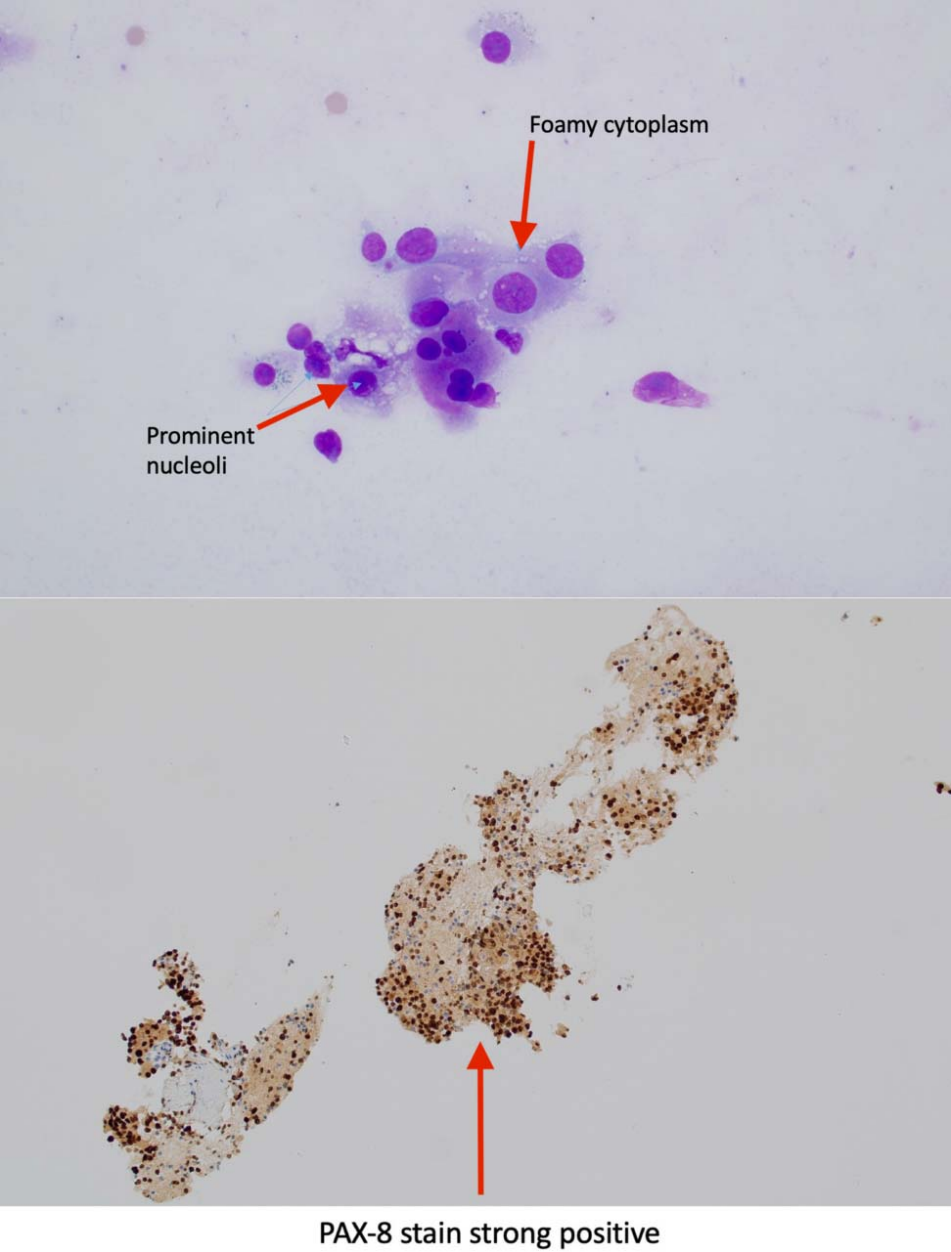
Histopathologic confirmation of metastatic RCC. The top panel shows a hematoxylin and eosin stain (×40 magnification) with foamy cytoplasm and prominent nucleoli. The bottom panel demonstrates strong PAX-8 immunohistochemical staining, consistent with metastatic RCC. RCC, renal cell carcinoma.

A 74-year-old man with a history of metastatic clear cell renal cell carcinoma (RCC), status postright nephrectomy and radiation therapy to para-aortic lymph nodes 10 years earlier, presented with progressive dysphagia over several months. His medical history included end-stage renal disease on peritoneal dialysis, coronary artery disease, and atrial fibrillation (post-Watchman device). Surveillance imaging had shown no disease recurrence until this presentation. Barium swallow indicated a midesophageal stricture (Figure 1). Chest computed tomography revealed an enlarging subcarinal mass in the posterior mediastinum, measuring 52 × 44 mm, compressing the esophagus (Figure 2). Esophagogastroduodenoscopy identified an ulcerated, malignant-appearing stenosis in the midesophagus (Figure 3). Endoscopic ultrasound-guided biopsy demonstrated an infiltrating mass with strong paired box gene 8 positivity, confirming metastatic RCC (Figure 4). The patient was referred to medical oncology to start systemic chemotherapy. Balloon dilation was performed to alleviate dysphagia with future plans for palliative stenting. This case underscores the potential for delayed gastrointestinal manifestations of RCC recurrence, exemplified by dysphagia due to esophageal compression from mediastinal metastasis, even a decade postnephrectomy. Such late recurrences, including isolated mediastinal lymph node metastases, have been documented in the literature.^1–3^ Clinicians should maintain a high index of suspicion for metastatic disease in patients with RCC presenting with new gastrointestinal symptoms, regardless of the time elapsed since initial treatment. Although isolated mediastinal metastases are uncommon, regular thoracic surveillance may be warranted in long-term follow-up.^4,5^

## DISCLOSURES

Author contributions: Writing manuscript and literature research: H. Ali; pathology expert: F. Poonam; supervision; supervision of case and manuscript: P. Mudireddy and article guarantor: H. Ali. Dr. Ali accepts full responsibility for the conduct of the study and the integrity of the work as a whole.

Financial disclosure: None to report.

Informed consent was obtained for this case report.
